# Intra- and inter-observer reliability of a new standardized diagnostic method using SPECT/CT in patients with osteochondral lesions of the ankle joint

**DOI:** 10.1186/s12880-016-0169-1

**Published:** 2016-12-07

**Authors:** Geneviève Hassink, Enrique A. Testa, André Leumann, Thomas Hügle, Helmut Rasch, Michael T. Hirschmann

**Affiliations:** 1 Department of Orthopaedic Surgery and Traumatology, Kantonsspital Baselland (Bruderholz, Liestal, Laufen), CH-4101 Bruderholz, Switzerland; 2 Department of Orthopaedic Surgery, University Hospital of Basel, Basel, Switzerland; 3 Institute of Radiology and Nuclear Medicine, Kantonsspital Baselland, CH-4101 Bruderholz, Switzerland

**Keywords:** SPECT/CT, Osteochondral lesions, CT, Computerized tomography, Scintigraphy, Ankle joint

## Abstract

**Background:**

Single Photon Emission Computed Tomography-Computed Tomography (SPECT/CT) gains an important part of diagnostics in patients with osteochondral lesions (OCL). SPECT/CT is a hybrid imaging modality, which combines a 3D scintigraphy (SPECT) and computerized tomography (CT) into one single procedure and combines metabolic data, structural and mechanical information.

The purpose of the study was to develop and evaluate a standardized method to anatomically localize and quantitatively analyze the bone SPECT tracer activity of the ankle joint using SPECT/CT.

**Methods:**

OCL on the talus were diagnosed in 16 patients by 99mTc-HDP-SPECT/CT and MRI by specialized orthopedic surgeons and radiologists and retrospectively included. The articular superior surface of the talus was subdivided in six anatomical regions (T1–T6).

Using customized software, absolute bone SPECT values for each anatomical area were analyzed. Relative bone tracer uptake was calculated in relation to specific reference regions representing bone SPECT tracer background activity. All measurements were performed twice by two independent observers, blinded to clinical information. Intraclass correlation coefficients (ICC) were calculated for inter- and intra-observer reliability. The intraclass correlation coefficients (ICC) showed an excellent inter- and intra-observer reliability.

**Results:**

The intraclass correlation coefficients (ICC) of all six regions are between 1.00 and 0.84 which is defined as very good. Results from region T1 to T6 impair slightly due to measurement regime.

All ICCs of observer 1 were nearly the same as the results of observer 2 in all regions.

**Conclusion:**

The presented standardized SPECT/CT algorithm is clinically feasible and showed high inter- and intra-observer reliability. It might help to better understand the complex pathology of OCL on the talar dome. The major potential benefit of SPECT/CT is the assessment of the subchondral bone plate and the subchondral bone.

## Background

Osteochondral lesions (OCL) of the ankle joint mostly affect the talus [1]. An unstable ankle joint, osseous malperfusion or malalignment might be predisposing factors for development of an OCL. Clinical diagnosis of OCL in the talus is challenging. Radiographs are the primary imaging in patients with ankle pain. In suspicion of an OCL CT and/or MRI are performed. MRI is considered as the gold standard of imaging for detection of OCL in the talus [2].

Recently, Single Photon Emission Computed Tomography/spiral Computed Tomography (SPECT/CT) has been recognized as promising imaging modality for patients with OCLs [3]. SPECT/CT combines the anatomical information from CT scans with metabolic information of SPECT in one single procedure. This allows an accurate assessment of the location and metabolic activity of an OCL or other pathologies and the surrounding bone. Buck et al. found that increased bone tracer uptake (BTU) can be considered to be a stronger marker for pain than bone marrow edema on MRI [4].

Furthermore, Hirschmann et al. [5] found a highly significant correlation between mechanical valgus/varus alignment and BTU of the medial and lateral compartment of the knee joint. The authors found that with the information provided by a SPECT/CT scan, the treatment of patients with chronic knee pain could be better targeted to the individual patient’s needs [5]. The intensity of BTU was associated with the severity and the localization of radiographic findings of osteoarthritis and overloading.

In another study it was shown that in patients with knee pain after total knee arthroplasty SPECT/CT is helpful to establish the diagnosis and guide further management [6].

The most frequently used tracer to detect increased bone metabolism is 99 m-Technetium-Hydroxymethylen-Diphosphonate (HDP), which is targeted towards active osteoblasts. Using this particular tracer, SPECT/CT, with its unique characteristics, is the only imaging modality allowing a diagnostic window into the subchondral bone plate, which is the most important area for development of OA as well as OCLs.

In previous studies, a significant correlation between BTU in SPECT/CT and pain scores in patients with OCLs of the ankle joint were reported [4, 7, 8]. Leumann et al. [9] also emphasized the fact that SPECT/CT is able to change the treatment proposed in patients with OCL on the talus. However, to date no correlation between clinical findings in the ankle joint and BTU was described.

The purpose of this study was to develop and evaluate a standardized measurement protocol for OCLs in the ankle joint. Similar standardization protocols for different clinical scenarios related to the knee joint were developed by Hirschmann et al. [10]. Their diagnostic SPECT/CT approach aims to allow reliable and reproducible 3D volumetric quantification of BTU in SPECT/CT.

## Methods

Sixteen patients aged between 18 and 76 years with an OCL on the talus (*n* = 16) diagnosed by experienced orthopedic surgeons and radiologists on MRI received a 99 m-Tc-HDP-SPECT/CT, performed between 2010 and 2012. The patients were prospectively collected and retrospectively included in this study. Inclusion criteria were age between 18 and 80 years and the confirmed diagnosis of an OCL on the talus, which was done by MRI. Exclusion criteria were acute talar or distal tibial fractures, previously diagnosed osteoarthritis, previous surgery to the ankle joint, kissing lesions, infectious disease, diabetes mellitus and osteoporosis.

Eight men and eight women with a mean age of 34 ± 17 years (range 20–76), with OCL (left : right 31% (*n* = 5) : 69% (*n* = 11)) were included (Table 1).

**Table 1:** Baseline characteristics of the included patients with OCL

	Sex (n)		Age (years)		Side (n)	
	Female	Male	Median	Range	Right	Left
total	8	8	34	20–76	11	5

All patients underwent 99 m-Tc-HDP-SPECT/CT imaging following a standardized protocol. All patients received a commercial 700 MBq (18.92 mCi) 99 m-Tc-HDP injection (Malinckrodt, Wollerau, Switzerland). SPECT/CT was performed using a hybrid system (Symbia T16, Siemens, Erlangen, Germany), which consists of a pair of low-energy, high resolution collimators and a dual-head gamma camera with an integrated 16-slice CT scanner (collimation of 16 × 0.75 mm). Planar scintigraphic images were taken in the perfusion phase (immediately after injection), the soft tissue phase (1 to 5 min after injection) and the delayed metabolic phase (2 h after injection). SPECT/CT was performed with a matrix size of 128× 128, an angle step of 32, and a time per frame of 25 s 2 h after injection.

**Fig. 1:**
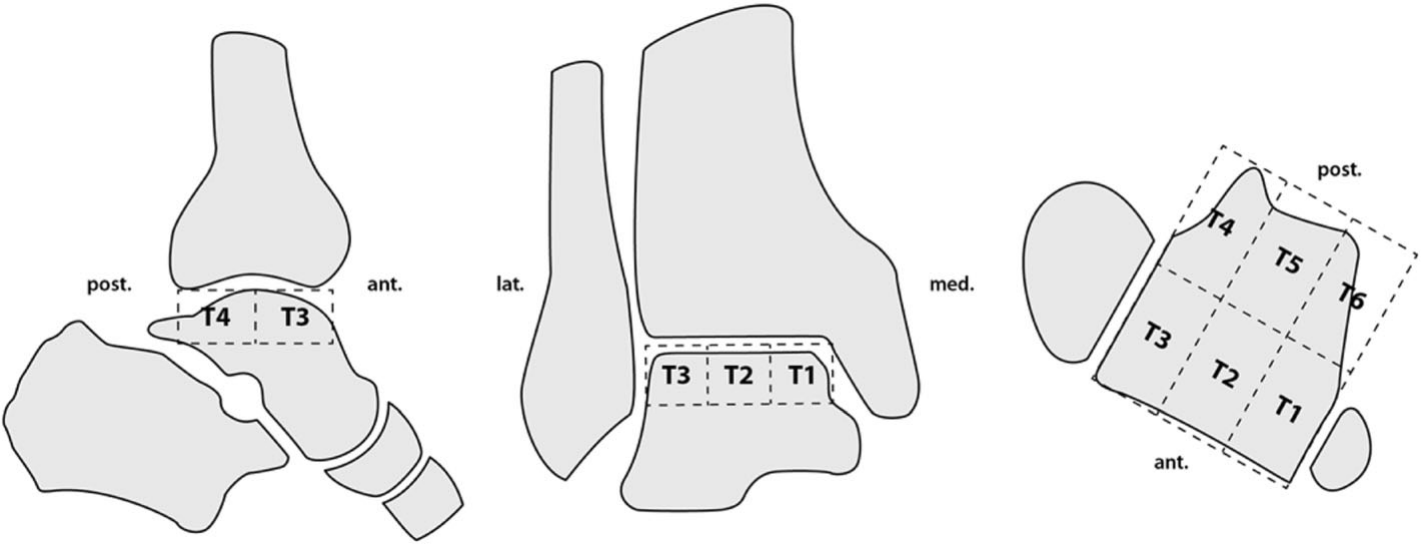
Scheme illustrating the measurement areas with subdivision in the 6 regions (T1-T6). On the *left* the sagittal view, in the *middle* the coronal view and on the *right* the axial view

The total BTU of the foot (global maximum) and the baseline bone activity in the proximal tibia were measured. The articular superior surface of the talus was then subdivided in six equally sized anatomical regions (T1–T6) (Fig. 1). Only the cartilage surface and the subchondral bone of the talus were included in the measurements. The volume of T1 was first measured and then applied to the following regions (T2–T6). The absolute maximum SPECT values (mean ± standard deviation) were analyzed using a customized software (IntroSPECT© v2.0, OrthoImagingSolutions, London, UK) [10] for each anatomical area separately and for the entire talus. With this software it is possible to measure BTU semi-automatically in anatomical regions.

**Fig. 2:**
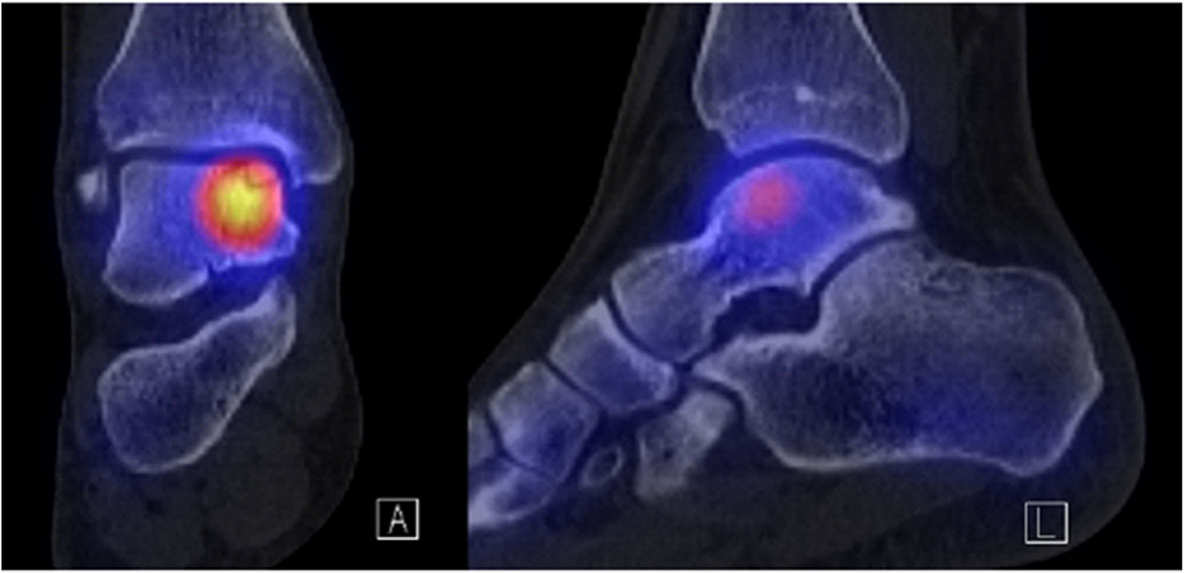
SPECT/CT of an ankle with an OCL on the talus dome. The *left* picture represents the coronal view, the *right* picture the sagittal view. Yellow indicates the highest activity in the bone, whereas in the tibia and the other foot bones the individual and physiological bone activity is shown as a *light blue*

**Fig. 3:**
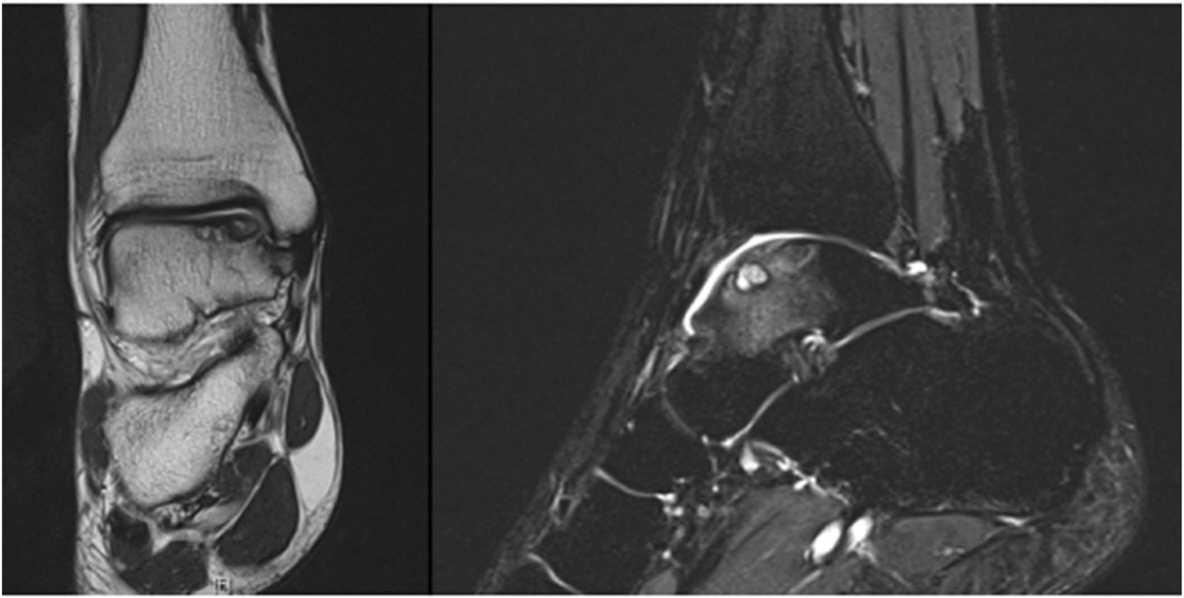
OCL at the medial talar dome is shown on MRI. *Left*: Proton-density-weighted coronal image shows a subchondral cystic area of the medial talar dome with adjacent cartilage defect. *Right*: T2-weighted fat-saturated sagittal image: Subchondral cystic lesion with surrounding bone marrow edema pattern of the talar body

**Fig. 4:**
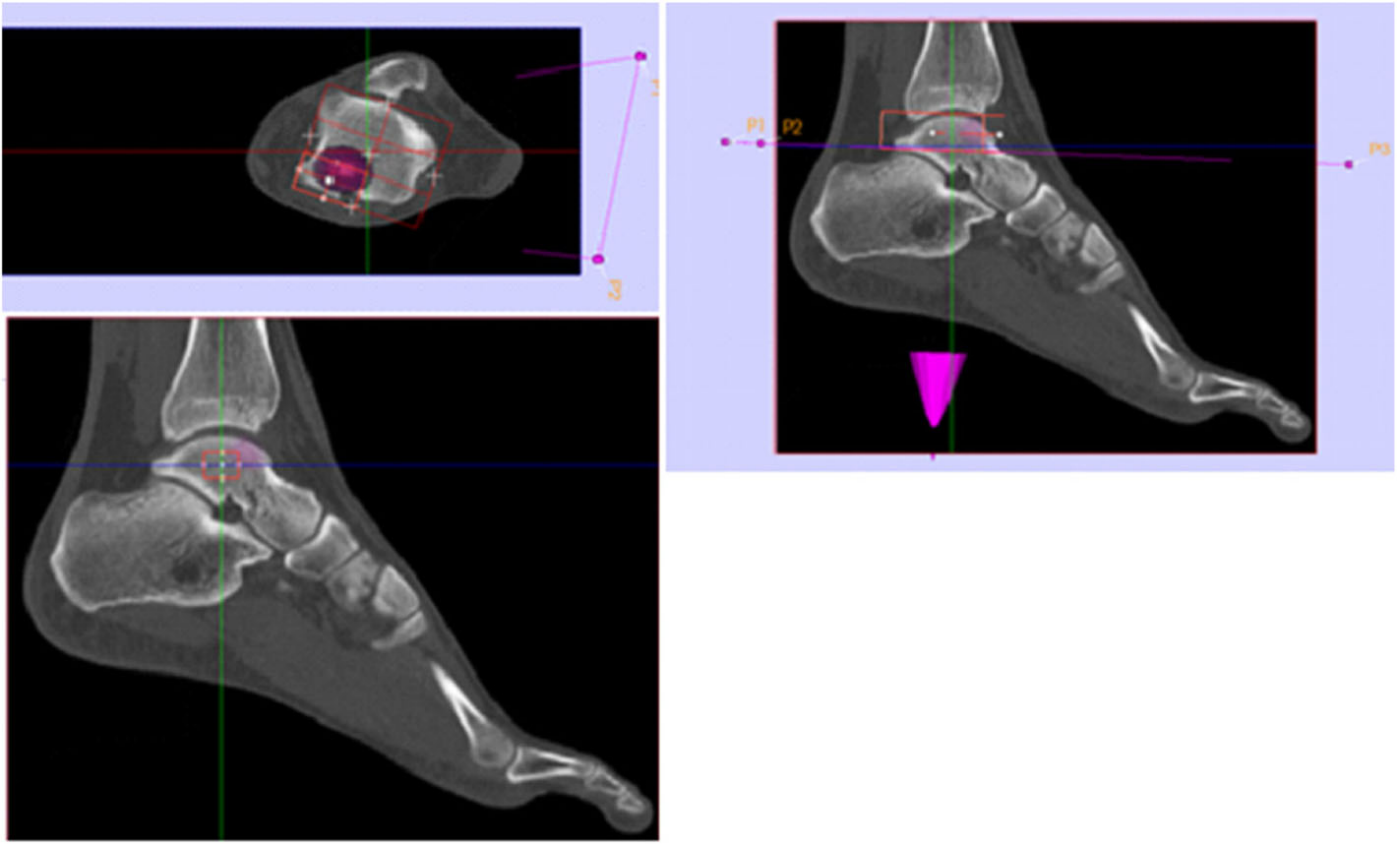
Illustration of BTU measurements with IntroSPECT©. *Top left*: Axial view of the talus surface. *Red* is the measurement *box*, placed in the six square grid for a measurement. The lesion is visible in *purple*. *Top right*: Sagital view with the center through the talar dome. The *red box* shows the measurement volume with the included bone. *Low left*: Sagital view. This time the *red box* includes only the center of the talus to make the last measurement in the talar center. In this measurement the volume is defined but not the position. *Top left*: Axial view on the talus surface with the measurement voxel box (*red*). The six squares divide the surface of the talus. *Purple* is the plane for adjustment of the patients foot position. *Green* and *red* are the plane which go through the talus. *Top right*: Sagittal view. *Red* is the measurement volume. It shows all the bone which is included into the measurement. *Low left*: Sagittal view. *Red* is the measurement volume (VOI) in the talus centre. The volume is defined, the position is not

Relative BTU was calculated in relation to specific reference regions representing the SPECT/CT tracer background activity. As reliable reference region the unaffected part of the proximal tibia was chosen. This baseline BTU allows the comparison within different patients by building ratios for every region of interest (Figs. 2, 3 and 4).

To evaluate the intra-observer reliability, two independent observers, blinded to clinical findings performed the measurements, with an interval of six weeks, twice on every patient. The observers were one experienced nuclear medicine specialist and one orthopedic surgeon. These were not aware of the other person’s and their own previous measurement results. The order of the measurements was chosen randomly.

Statistical analysis was performed by an independent professional statistician. Data were analysed using SPSS 16.0 (SPSS, Chicago, U.S.A.). Descriptive statistics noted the absolute and relative BTU in each anatomical area. All the measurements were compared using intraclass correlation coefficients (ICC) for inter- and intra-observer reliability (Tables 2 and 3).

**Table 2:** Intra- and inter-observer-reliability. In the upper part the local absolute and the maximum of every region is shown whereas in the lower part the ratio to the individual physiological bone activity (measured in the not affected proximal tibia) is listed

	Intra observer 1			Intra observer 2			Inter R1–R2		
	ICC	Lower	Upper	ICC	Lower	Upper	ICC	Lower	Upper
Total Uptake (Global Max)	1	1	1	1	1	1	1	1	1
Baseline Activity (Tibia)	1	0.99	1	1	1	1	0.98	0.94	0.99
Local (max) T1	1	1	1	1	1	1	1	0.99	1
Local (max) T2	0.99	0.98	1	1	0.99	1	0.99	0.98	1
Local (max) T3	0.99	0.98	1	1	1	1	1	0.99	1
Local (max) T4	0.98	0.93	0.99	0.97	0.91	0.99	0.98	0.95	0.99
Local (max) T5	0.96	0.89	0.99	0.96	0.9	0.99	0.98	0.95	0.99
Local (max) T6	0.99	0.97	1	0.99	0.97	1	0.97	0.92	0.99
Local (max) Talus Centre	0.96	0.89	0.99	0.96	0.89	0.99	0.98	0.96	0.99
Ratio T1	1	0.99	1	1	1	1	0.99	0.98	1
Ratio T2	0.99	0.98	1	0.99	0.98	1	0.98	0.94	0.99
Ratio T3	0.98	0.95	0.99	1	0.99	1	0.98	0.95	0.99
Ratio T4	0.96	0.9	0.99	0.94	0.83	0.98	0.94	0.84	0.98
Ratio T5	0.97	0.92	0.99	0.93	0.81	0.97	0.98	0.93	0.99
Ratio T6	1	0.99	1	0.99	0.97	1	0.94	0.84	0.98
Ratio Talus Centre	0.94	0.84	0.98	0.94	0.84	0.98	0.97	0.92	0.99

**Table 3:** A measurement example of a patient by a single observer with absolute values in two different measurement trials. Measurements of the BTU in a talus with an OCL on the anteromedial surface

			anterior					
	Global Maximum Uptake	Base line (tibia)	Medial (T1)		Intermediat (T2)		Lateral (T3)	
			local (max)	ratio	local (max)	ratio	local (max)	ratio
Patient 1, Measurement 1	597	76	597	7.86	286	3.76	126	1.66
Patient 1, Measurement 2	597	74	597	6.86	484	5.56	138	1.59
Patient 1, Measurement 1	153	2.01	523	6.88	420	5.53	132	1.74
Patient 1, Measurement 2	191	2.2	384	4.41	312	3.59	132	1.52
	local (max)	ratio	local (max)	ratio	local (max)	ratio	local (max)	ratio
	Talus Centre		Medial (T6)		Intermediat (T5)		Lateral (T4)	
			posterior					

An ICC of 1.00 was defined as excellent whereas the ICC from 0.81 to 1.00 is an almost perfect, from 0.61 to 0.80 a substantial, from 0.41 to 0.60 a moderate, from 0.21 to 0.40 a fair and from 0.0 to 0.20 a slight result [11].

## Results

The ICC for each anatomical region is presented in Tables 2 and 3. The following examples show the interpretation of the results for regions T1 and T6. As seen in Tables 2 and 3 the ICC of observer 1′s measurement of the global maximum was 1.00 (CI 1.00–1.00) and the ICC of the baseline activity was 1.00 (CI 0.99–1.00). The ICC for the inter-observer reliability was 1.00 (CI 1.00–1.00) for the global maximum and 0.98 (CI 0.94–0.99) for the baseline activity.

For T1 the ICC of the local maximum of observer 1′s measurements was 1.00 (CI 1.0–1.0). The ICC for the ratio, which was calculated between the local maximum and the individual baseline bone activity of the patients was 1.00 (CI 0.99–1.0). The inter-observer reliability for T1 was 1.00 (ICC 0.99–1.00) for the local maximum and 0.99 (CI 0.98–1.00) for the ratio.

All ICCs of observer 1 were nearly the same as the results of observer 2.

For T6 the ICC of the local maximum of observer 2′s measurement was 0.99 (CI 0.97–1.00). The ICC result for observer 2′s ratio of T6 is 0.99 (CI 0.97–1.00). The inter-ratio of this area was 0.97 (CI 0.92–0.99) for the local maximum and 0.94 (CI 0.84–0.98) for the ratio.

For the talus centre the ICC of the local maximum of observer 1 was 0.96 (CI 0.89–0.99) and 0.94 (CI 0.84–0.98) for the ratio. The inter-observer reliability was 0.98 (CI 0.96–0.99) for the local maximum and 0.97 (CI 0.92–0.99) for the ratio.

## Discussion

The most important findings of the present study were: Firstly, this study obtained an excellent inter- and intra-observer reliability of the new SPECT/CT measurement method. The intraclass correlation test (ICC) showed values >0.9, which are defined as almost excellent [11].

A considerable number of limitations have to be acknowledged. One is the foot position during SPECT/CT scanning. Lack of a standardized foot position results in different projection angles due to more or less plantar-flexion in the ankle joint. In the protocol used this is dealt by establishing a standardized measurement plane through the talus according to the foot position. However, the procedure itself is challenging. Difficulties occur especially with high plantar flexion of the patient’s foot. For further studies the position of the foot should be standardized using a positioning device. The use of differently sized pillows or foot splints could be helpful for that purpose. These devices should aim for a ninety degrees angle between talus/foot and tibia. In this position the talus is perpendicular and in the measuring plane.

Secondly, more OCL lesions were seen in the medial than the lateral talus. A similar distribution of OCLs on the talus surface has been described by Raikin et al. [12] analyzing 424 patients with OCLs on the talus. In the area of T1 and T6 (Fig. 1), which represents the medial part of the talus, the maximal tracer values are higher, compared to the values in T3 and T4, which represent the lateral parts. Intermediate parts, which are represented by T2 and T5, are the areas with the lowest tracer activity in this study. A patient’s example with absolute values is shown in Table 3.

This typical distribution might be attributed to the injury mechanism which leads to an OCL on the talar dome. Takao et al. [13] described the distribution of OCLs on the talar dome with regards to their trauma. Four groups according the injury mechanism were described: supination-adduction (SA), supination-eversion (SE) the most common injury, pronation-abduction (PA) and pronation-eversion (PE), the rarest injury. Different trauma result in different locations of OCL on the talus dome. Most of the OCL occur on the medial dome, resulting from a SE-trauma. Takao et al. reported that subacute lesions are often only involving the cartilage (surface), whereas chronic injuries involve the subchondral bone plate of the joint. According to Takao et al. the deeper the defect, the more complicated the therapy is and the more painful the lesion is. A fact which is related to the subchondral bone plate, which has many nerve endings, compared to the nerve free tissue in the cartilage.

Thirdly, this new measurement protocol and method is reliable. The new protocol was developed and two observers analyzed a considerable number of SPECT/CTs from different ankle joints. Both observers pointed out the user friendliness and handiness of the program and the simplicity of the application in their evaluation. Nevertheless the low case number and the lack of a control group is a limitation and has to be considered in a further evaluation.

A strength of this study is that the proposed measurement protocol could be used for further studies about OCL on the talus or for other pathologies in the ankle joint. Valderrabano et al. [14] found that ligamentous injury and OCL are a predisposing factor in the development of osteoarthritis in the ankle joint. In this case an examination of the SPECT/CT with the new measurement protocol could help to quantify the higher metabolism on the lateral or medial part of the talus surface and to diagnose the OCL earlier to prevent or to slow down the progression to osteoarthritis.

The quantitative and standardized measurement protocol gives the possibility to measure the SPECT/CT BTU within the subchondral bone plate. As described above the subchondral bone plate often is the cause of chronic pain due to the high distribution of nerve endings. Especially in the ankle joint, compared with the knee joint, the subchondral bone plate is often affected by cysts. As the repair mechanism for chondral and subchondral defects is coming from the subchondral bone (bone marrow with stem cells, blood clot formation), cartilage defects are much likely to heal in patients with an subchondral and not only a chondral defect. This matter and the importance of the subchondral bone plate in the development of osteoarthritis out of an OCL was pointed out by Madry et al. [15] in their work. Furthermore they emphasized the importance of taking the subchondral bone plate into account when deciding over a treatment strategy after a joint injury with a chondral or subchondral defect.

In a further study the subchondral bone plate should be measured separately to get a more profound insight into the pathology of OCL.

Furthermore a correlation between the distribution or the level of BTU and different pathologies has yet not been examined. However, this could be an interesting approach for further studies.

## Conclusions

The presented standardized SPECT/CT algorithm is clinically feasible and showed a high inter- and intra-observer reliability. It might help to better understand the complex pathology of OCL on the talar dome. The major potential benefit of SPECT/CT is the assessment of the subchondral bone plate and the subchondral bone.

## Abbreviations

BTU: Bone tracer uptake
CI: Confidential interval
ICC: Intraclass correlation test
OCL: Osteochondral lesion
SPECT/CT: Single photon emission computed tomography-computed tomography
